# A decade of e-cigarettes: trends and determinants among U.S. middle and high school students (2013–2022)

**DOI:** 10.1186/s12889-025-25719-0

**Published:** 2025-12-04

**Authors:** Khuloud Almugbel, Shaikha K. Aldukhail

**Affiliations:** 1 BDS, MSD, CAGS Dental Public Health, Princess Nourah Bint Abdulrahman University, P.O. Box 84428, Riyadh, 11671 Saudi Arabia; 2 Department of Preventive dental sciences, College of dentistry, Princess Nourah bint Abdulrahman University, P.O. Box 84428, Riyadh, 11671 Saudi Arabia

**Keywords:** Vape, Electronic cigarette, E-cigarette, Tobacco, E-hookah, Adolescent health, Electronic nicotine delivery systems

## Abstract

**Objectives:**

To analyze the 10-year trend (2013–2022) in e-cigarette use among U.S. middle and high school students, identify reasons for use across demographic subgroups, and explore factors associated with ever using e-cigarettes.

**Methods:**

Cross sectional data from the National Youth Tobacco Survey (NYTS) from 2013 to 2022 were used to study e-cigarette use trends among middle and high school students. Descriptive, multivariable logistic regression, and joinpoint models analyzed factors associated with ever using e-cigarettes. Statistical analyses were conducted using SAS and Python, with a significance level of *p* < 0.05 taking into account weighting/ complex sampling.

**Results:**

E-cigarette use among students peaked in 2019 and then declined from 2020 to 2022. Usage was highest among Non-Hispanic White and Hispanic students, and lowest among Asian students. In 2022, females comprised over half of students who ever used e-cigarettes (53%). The main reasons for using e-cigarettes included influence from friends/family, appealing flavors, and the ability to use e-cigarettes discreetly. Males, Non-Hispanic Whites, high school students, and those with a history of cigarette use had higher odds of ever using e-cigarettes in the US from 2013 to 2022. Joinpoint regression revealed a significant increase in use among males from 2014 to 2019 and among females from 2015 to 2019 (*p* < 0.05). Similarly, a significant upward trend was observed after 2014, with the AAPC ranging from approximately 183% in White students to 330% in Asian students.

**Conclusions:**

Our findings indicate that between 2013 and 2019, adolescents in the U.S. experienced a significant upward trend in e-cigarette use. Non-Hispanic White adolescents were the predominant adolescents who use e-cigarettes, followed by Hispanic adolescents. The study suggests this trend could be driven by peer influence and product flavors. However, in 2022, females represented over half of adolescents who had ever used e-cigarettes, indicating a shift in use patterns over time. These results underscore the importance of policy interventions to address e-cigarette use among youth in this population.

## Background

Electronic cigarette (E-cigarette), also known as vapes or e-hookah, are battery-powered devices designed to simulate smoking by producing an aerosol [1]. They come in various forms, such as pens, flashes, tank systems, or disposables, and typically contain nicotine, flavorings, and other chemicals [2, 3]. E-cigarette use among adolescents has emerged as a pressing public health concern, garnering increasing attention from researchers, policymakers, and healthcare professionals. E-cigarettes were initially marketed in 2007 as smoking cessation aids and a safer alternative to conventional cigarettes [1]. However, they gradually became the most used tobacco product, particularly among U.S. youth who may have never engaged in cigarette smoking [1]. In the United States, an estimated 1.63 million middle and high school students were currently using e-cigarettes in 2024 [1]. In 2018, the Surgeon General’s report emphasized the urgent need to address the youth e-cigarette epidemic, taking swift action to protect children from nicotine addiction and associated health risks [4]. While the prevalence of cigarette smoking has shown a decline in the USA from 42.4% in 1965 to 12.5% in 2020 [5], the dual use of e-cigarettes alongside other tobacco products remains concerning [1]. In 2020, about one in three high school students (36.8%) who vaped also used other tobacco products, and among middle school students, this rate was even higher at one in two (49.0%) [1, 6]. The prevalence of e-cigarette use among middle and high school students saw a remarkable 900% increase from 2011 to 2015 [7].

This increasing prevalence has intensified concerns regarding the potential health consequences of e-cigarette use, particularly among adolescents. E-cigarettes have the potential to deliver addictive substances like nicotine and marijuana [8]. There are many reported health hazard cases associated with e-cigarettes such as pneumonia, diffuse alveolar hemorrhage, multiple reactive pulmonary nodules, subacute bronchiolitis, oral and tongue injuries, dental injuries, facial fractures, thermal injuries, and fatal intoxication [9]. E-cigarette use is associated with increased risk of periodontal disease and caries, partly through promoting *Staphylococcus aureus* colonization and oral inflammation [10, 11]. E-cigarettes may also contribute to the development of recurring oral ulcers and erythema multiforme, as documented in several case reports [12, 13]. However, the long-term effects of e-cigarette use on both oral and systemic health remain under investigation.

Evidence have shown that sex and race significantly influence e-cigarette use, reflecting a shift from historical smoking trends where combustible cigarette use was predominantly common among males [14–16]. For example, a study based on the Utah Department of Health Prevention Needs Assessment (PNA) survey found that American Indian or Alaskan Native, Black or African American, Hispanic/Latino, Multiracial, and Native Hawaiian or other Pacific Islander youth had significantly higher odds of both lifetime and past 30-day vaping compared to non-Hispanic White youth [15]. Moreover, recent literature has highlighted emerging sex differences in vaping patterns, with evidence suggesting increased uptake among adolescent females [14, 16]. Although these disparities have been documented, few studies have systematically examined how these differences have evolved over time. Particularly across a decade marked by rapid shifts in product design, marketing strategies, regulatory changes, and evolving cultural norms. Addressing this gap is essential for informing targeted prevention and policy interventions, as trends may reflect shifting marketing strategies, differential access or exposure, or sociocultural influences across groups. Therefore, the objectives of this study were to [1] examine the 10-year trend (2013–2022) of ever e-cigarette use among U.S. middle and high school students [4], describe reasons for e-cigarette use across demographic subgroups, and [5] assess factors associated with ever e-cigarette use among U.S. adolescents.

## Materials and methods

This cross-sectional study utilized data from the National Youth Tobacco Survey (NYTS), which is publicly accessible and offers nationally representative information on tobacco-related beliefs, attitudes, and behaviors among middle and high school students [17]. We analyzed data from the NYTS cycles spanning 2013 to 2022 [18], as these were the only cycles incorporating questions related to e-cigarettes at the time of the analysis in 2023. In the Survey [17], e-cigarettes are defined as " battery-powered devices that usually contain a nicotine-based liquid that is vaporized and inhaled, such as JUUL, Vuse, blu, and Logic. It is also known as e-cigs, vape-pens, e-hookahs, vapes, or mods”.

Our primary dependent variable utilized the e-cigarette questions; which assessed if the participants ever use “Have you ever used an e-cigarette, even once or twice?” (Yes/No). We analyzed the reasons for e-cigarette use among individuals who have ever used them using the question: “What are the reasons for using e-cigarettes?“. Answers included: (Friend or family member used them, To try to quit using other tobacco products, such as cigarettes, They cost less than other tobacco products, such as cigarettes, They are easier to get than other tobacco products, such as cigarettes, Famous people on TV or in movies use them, They are less harmful than other forms of tobacco, such as cigarettes, They are available in flavors, such as mint, candy, fruit, or chocolate, They can be used in areas where other tobacco products, such as cigarettes, are not allowed/can use them unnoticed at home or at school, Other reason), participant could select multiple options.

The primary covariates analyzed in this study included sex (male, female), grade level (middle school, high school), and race/ethnicity (non-Hispanic White, non-Hispanic Black, Hispanic, non-Hispanic Asian, and other races; including American Indian or Alaska Native, Native Hawaiian or Other Pacific Islander, and Multiple Races), tobacco cigarette use status categorized as; never smoked, experimental smokers (less than 100 cigarettes in a lifetime), established smokers (more than 100 cigarettes in a lifetime). Weighted percentages, prevalence and unweighted frequencies were reported to describe e-cigarette use, and reasons for e-cigarette use. Multivariable logistic regression models were fitted to measure factors associated with ever e-cigarettes use (among all participants). The reported adjusted odds ratios (aORs) and 95% confidence intervals (95% CIs) were adjusted for relevant demographic covariates. Survey weights provided by the CDC were applied to account for complex sampling design, differential probabilities of selection, nonresponse, and post-stratification adjustments by sex, grade, and race/ethnicity allowing for nationally representative estimates and valid variance estimation. Further details on the NYTS survey weighting methodology are available in the CDC’s methodological documentation [18]. Joinpoint regression analysis was conducted using calendar year as the independent variable and the prevalence of ever e-cigarette use as the dependent variable, with a log-linear model and Poisson error structure to estimate Annual Percent Change (APC) and Average Annual Percent Change (AAPC); model selection was based on the permutation test to identify statistically significant joinpoints (*p* < 0.05). All statistical analyses utilized SAS V.9.4 statistical software (SAS Institute, Cary, N.C), and statistical significance was set at *p* < 0.05.

## Results

### Trends in e-cigarette use among middle and high school students -US (2013–2022)

In this study, we analyzed data from the (NYTS) covering the period from 2013 to 2022 (*N* = 199,113). Table 1 presents the characteristics of adolescents who have ever used e-cigarettes. The majority of students who used e-cigarettes during this period (2013–2022) were high school students. The percentage of adolescents who had ever used e-cigarettes increased gradually among males from 2013 to 2021. However, in 2022, females represented over half (53%) of adolescents who had ever used e-cigarettes. The prevalence of adolescents who had ever used e-cigarettes and were people who smoke cigarettes decreased over the decade (2013–2022). Approximately one-third of adolescents who had ever used e-cigarettes in 2013 (33%) were people who smoke cigarettes, while this prevalence dropped to 9% by 2022. Conversely, among adolescents who had ever used e-cigarettes, the percentage who had never smoked cigarettes increased from 14% in 2013 to 52% in 2018 (Table 1). Non-Hispanic White adolescents comprised the majority of adolescents who had ever used e-cigarettes from 2013 to 2022, followed by Hispanic adolescents, who accounted for 20% in 2013 and 27% in 2022.

**Table 1:** Prevalence of Ever E-Cigarette Use Among Middle and High School Students - US (2013–2022)

**Demographics**	**2013n(%)**	**2014n(%)**	**2015n(%)**	**2016n(%)**	**2017n(%)**	**2018n(%)**	**2019n(%)**	**2020n(%)**	**2021n(%)**	**2022n(%)**
Sex										
MalE	785(57.26%)	2355(56.50%)	2570(55.14%)	2452(54.26%)	1953(54.26%)	2662(52.39%)	3426(52.83%)	1850(51.94%)	1755(50.59%)	2798(47.28%)
Female	586(42.74%)	1813(43.50%)	2075(44.86%)	1996(45.74%)	1704(45.74%)	2365(47.61%)	2958(47.17%)	1855(48.06%)	1891(49.41%)	2973(52.72%)
GradE										
Middle	267(19.63%)	1150(27.32%)	1181(22.40%)	1227(24.87%)	828(22.48%)	1138(20.80%)	1783(25.23%)	766(17.67%)	768(16.43%)	1256(18.75%)
High School	1093(80.37%)	3059(72.68%)	3484(77.60%)	3251(75.13%)	2852(77.52%)	3924(79.20%)	4617(74.77%)	2943(82.33%)	2887(83.57%)	4537(81.25%)
Race										
NH-White	716(62.13%)	1792(54.41%)	2046(52.41%)	1935(55.09%)	1769(58.48%)	2733(60.58%)	3161(56.47%)	1965(54.34%)	1917(57.41%)	2653(53.36%)
NH-Black	114(6.70%)	443(10.29%)	483(10.19%)	459(8.24%)	364(7.22%)	311(6.33%)	586(9.42%)	196(6.56%)	385(7.56%)	500(9.57%)
Hispanic	320(19.96%)	1308(24.80%)	1520(27.79%)	1436(28.05%)	1060(25.06%)	1373(22.95%)	1902(25.06%)	1196(28.53%)	903(23.48%)	1524(26.87%)
NH-Asian	27(2.26%)	102(1.89%)	79(1.80%)	93(1.71%)	82(1.64%)	107(2.80%)	162(2.19%)	93(2.76%)	82(2.70%)	172(2.05%)
Other Races*	155(8.95%)	383(8.19%)	381(7.82%)	404(6.90%)	285(7.59%)	388(7.34%)	491(6.85%)	232(7.81%)	320(8.84%)	872(8.15%)
Cigarette use										
Never	184(13.61%)	1405(34.04%)	1974(42.25%)	1902(49.82%)	1686(47.52%)	2548(51.98%)	872(33.35%)	378(29.87%)	387(34.02%)	660(35.09%)
Experimental**	723(53.48%)	2153(52.16%)	2156(47.69%)	1424(39.83%)	1646(44.05%)	2030(39.85%)	1231(51.43%)	724(57.32%)	604(56.48%)	1031(55.78%)
Established***	447(32.91%)	570(13.81%)	451(10.06%)	337(10.35%)	312(8.43%)	431(8.17%)	330(15.21%)	148(12.80%)	115(9.50%)	185(9.13%)

e-cigarette ever use: has smoked ≥ 1 puff of an e-cigarette

All variables are reported with unweighted frequency and weighted percentage to account for complex survey design

*Other Races= (American Indian or Alaska Native, Native Hawaiian or Other Pacific Islander, and Multiple Races)

**Experimental = less than 100 days

***Established = more than 100 days

**Fig. 1:**
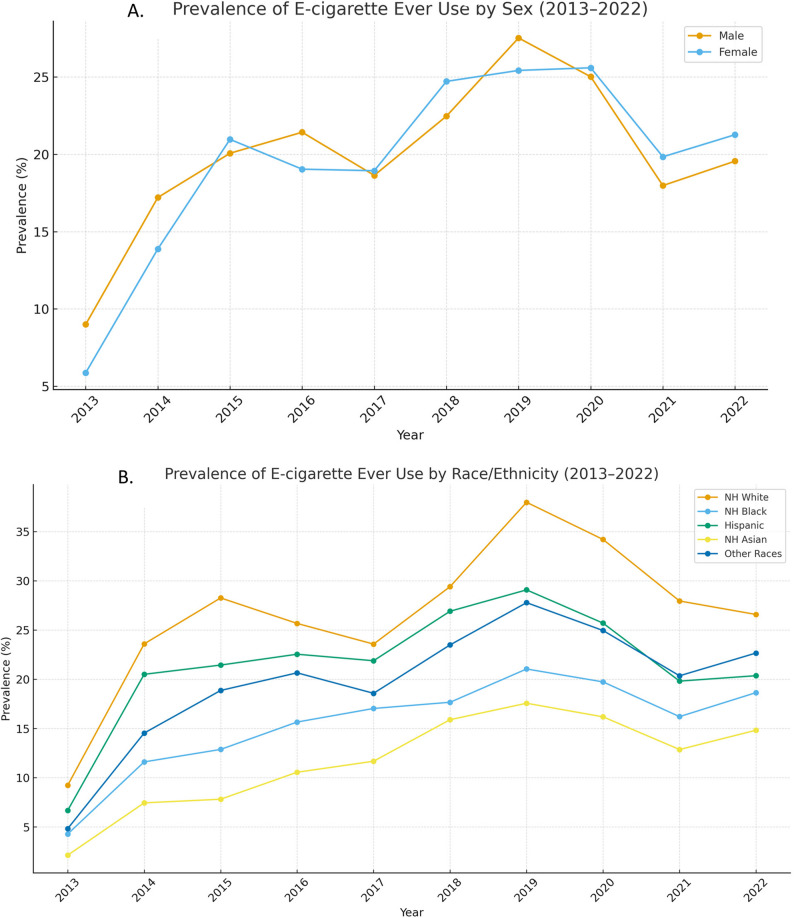
Trends of e-cigarette ever use among middle and high school students (2013–2022). Panel **A**: Trends stratified by sex (male vs female). Panel **B**: Trends stratified by race (White, Black, Hispanic, Other).“Other” includes American Indian or Alaska Native, Native Hawaiian or Other Pacific Islander, and Multiple Races

The trends in e-cigarette use among middle and high school students were explored, stratified by sex and race (Fig. 1). Over the decade, the prevalence of e-cigarette ever use increased among both males and females, peaking in 2019 at approximately 35% for males and 33% for females. Meanwhile in 2013, about 9% of males and 6.6% of females had ever used e-cigarettes. The prevalence of e-cigarette ever use among Non-Hispanic White students doubled from 9.2% in 2013 to 20.5% in 2014, reaching an all-time high of 37.1% in 2019. A similar trend was observed among Non-Hispanic Black students, with ever-use increasing from 4% in 2013 to 14.2% in 2014, and about a quarter of Non-Hispanic Black students (25.7%) reported ever trying the product by 2019. The prevalence among Hispanic students increased from 8% in 2013 to 32% in 2015. The prevalence of e-cigarette use was highest among those identifying as other races and lowest among Asian students throughout the analyzed decade. The trend across all subgroups continued to rise until 2019, after which it showed a decrease from 2020 to 2022 (Fig. 1).

### Exploring the reason for e-cigarettes ever use among middle and high school students- US (2013–2022)

The most common reasons for e-cigarette use among middle and high school students across the decade from 2013 to 2022 were the use of the product by friends or family members, its availability in flavors such as mint, candy, fruit, or chocolate, the ability to use it in areas where other tobacco products, like cigarettes, are not allowed, and the possibility of using it unnoticed at home or at school (Table 2). In 2020, two thirds of adolescents (66%) reported ever using e-cigarettes due to the influence of a friend or family member. This reason for use experienced a sharp drop to only a quarter of them (25%) citing this reason in 2021 and 2022. Over the decade, fewer adolescents believed that e-cigarettes were less harmful than traditional cigarettes, with 47% holding this belief in 2015, 30% in 2018, and only 12% in 2022. Additionally, the availability of appealing flavors like mint, candy, fruit, or chocolate was a significant motivator for e-cigarette use, particularly in the early years from 2015 to 2019.

**Table 2:** Reasons for Ever E-Cigarette Use Among Middle and High School Students - US (2013–2022)

**Reason for Use**	*2013n(%)*	*2014n(%)*	*2015n(%)*	*2016n(%)*	*2017n(%)*	*2018n(%)*	*2019n(%)*	*2020n(%)*	*2021n(%)*	*2022n(%)*
Friend or family member used them	-	-	2212(63.83)	1756(54.78)	1596(57.27)	2100(55.62)	1970(48.11)	1842(66.06)	430(25.25)	780(25.53)
To try to quit using other tobacco products, such as cigarettes	-	-	351(14.13)	287(14.17)	222(9.47)	362(11.81)	323(11.86)	141(9.19)	61(6.08)	84(4.00)
They cost less than other tobacco products, such as cigarettes	-	-	240(8.98)	153(6.19)	125(5.11)	197(6.48)	225(8.38)	80(4.34)	65(6.23)	120(5.59)
They are easier to get than other tobacco products, such as cigarettes	-	-	-	231(9.30)	165(7.21)	248(8.23)	329(11.58)	154(8.97)	87(7.96)	171(7.98)
Famous people on TV or in movies use them	-	-	124(4.83)	86(3.31)	78(3.05)	90(2.68)	268(9.24)	111(6.20)	46(3.84)	83(4.15)
They are less harmful than other forms of tobacco, such as cigarettes	-	-	1331(47.33)	799(28.67)	614(26.50)	1004(30.46)	968(31.05)	373(21.26)	143(13.42)	227(11.79)
They are available in flavors, such as mint, candy, fruit, or chocolate	-	-	1957(61.55)	1360(46.96)	1031(41.39)	1540(45.29)	1349(41.37)	626(31.87)	194(17.14)	374(18.04)
They can be used in areas where other tobacco products, such as cigarettes, are not allowed/can use them unnoticed at home or at school	-	-	518(20.35)	283(12.64)	146(6.05)	262(8.40)	814(28.94)	436(24.12)	181(17.08)	395(20.04)
Other reason	-	-	1318(45.24)	1370(46.78)	965(39.04)	1384(40.57)	866(28.57)	617(33.12)	274(23.88)	475(23.08)

(-) Data was not collected that year

All variables are reported with unweighted frequency and weighted percentage to account for complex survey design

**Fig. 2:**
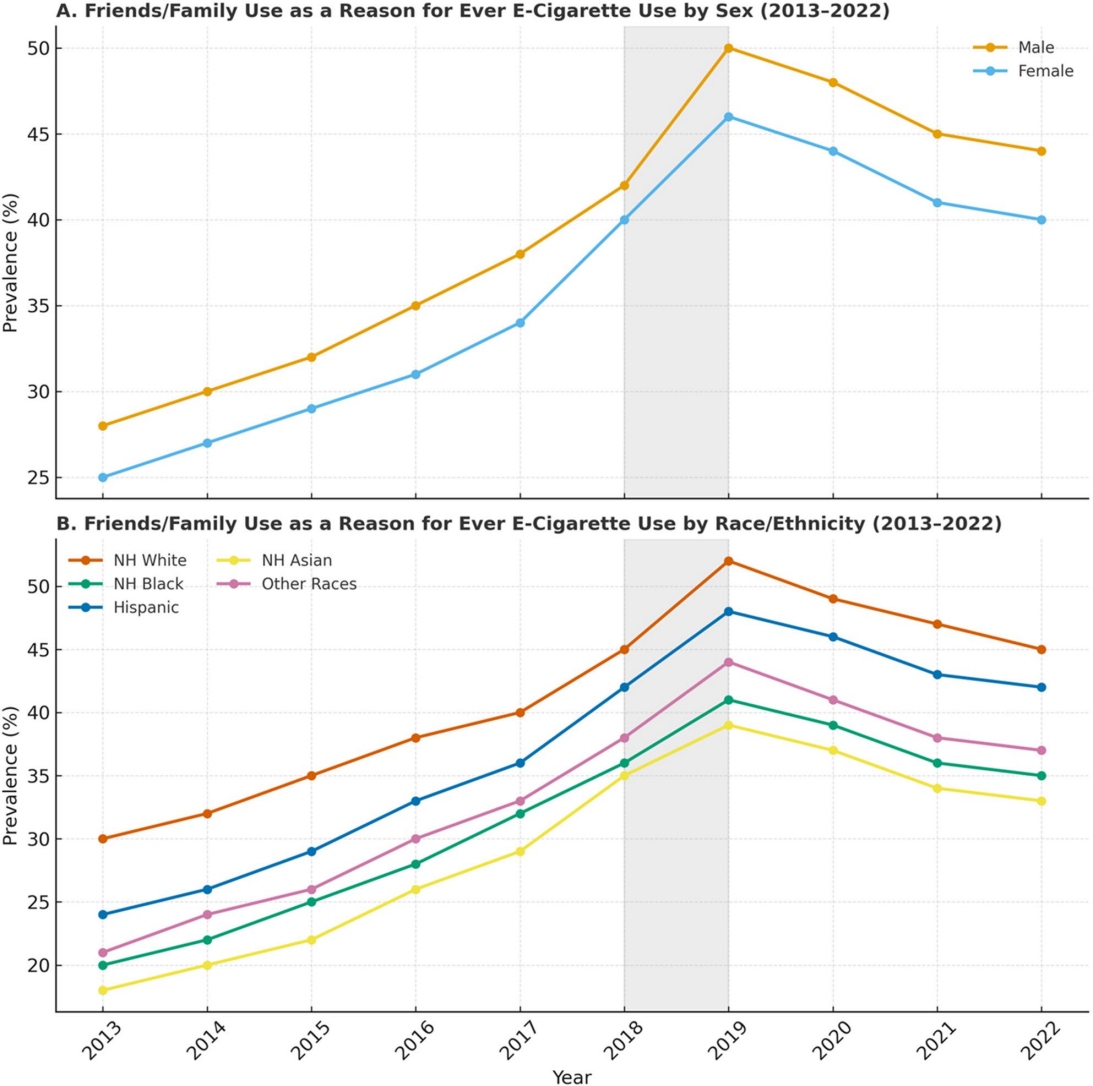
Analysis of the reason “Friends or family use” for ever e-cigarette use among U.S. middle and high school students (2013–2022). Panel **A**: Proportion reporting “friends or family use” as a reason for vaping, stratified by sex. Panel **B**: Proportion reporting “friends or family use” as a reason for vaping, stratified by race/ethnicity

**Fig. 3:**
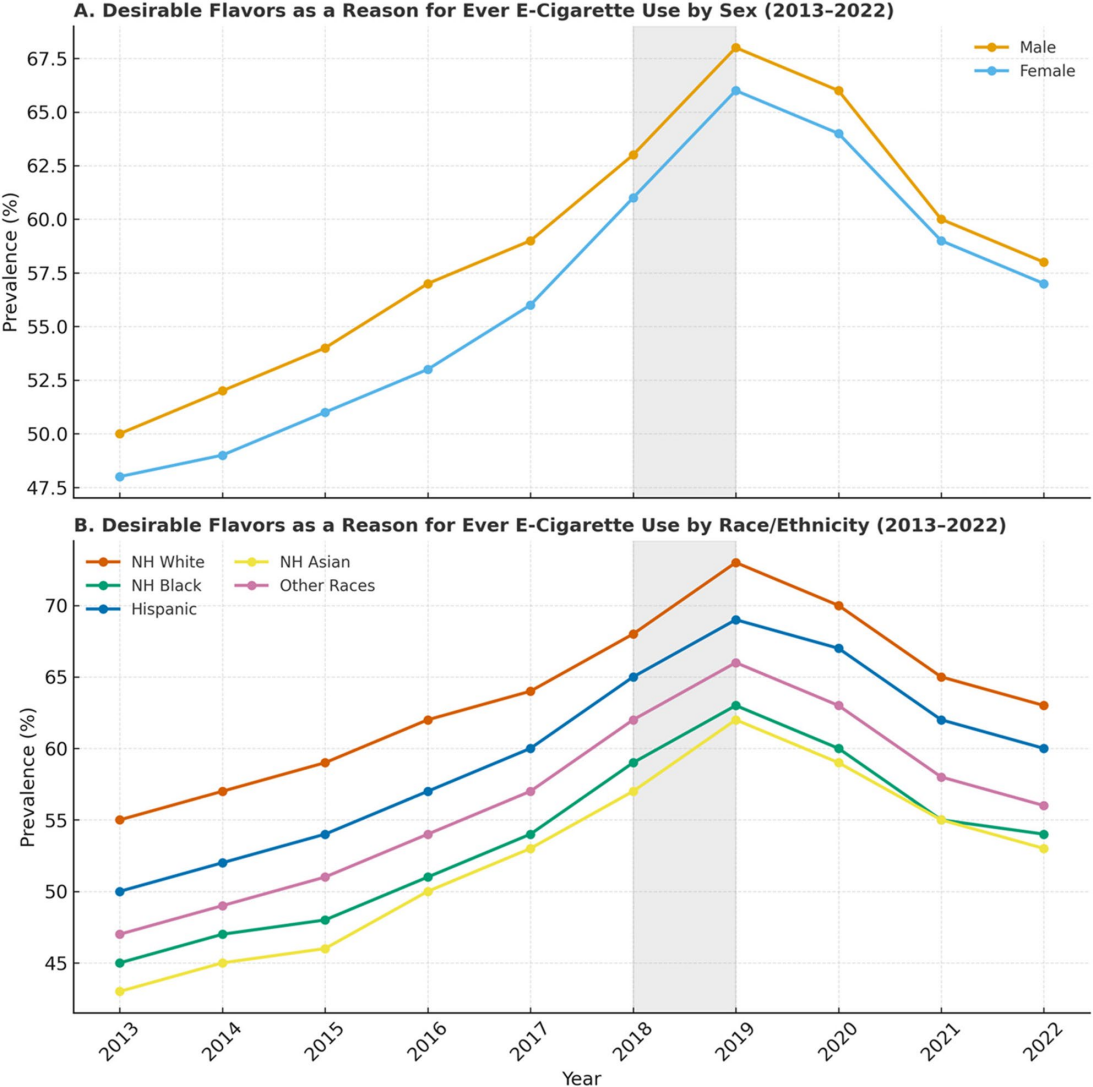
Analysis of the reason “Desirable flavors” for ever e-cigarette use among U.S. middle and high school students (2013–2022). Panel **A**: Proportion citing “desirable flavors” as a reason for use, stratified by sex. Panel **B**: Proportion citing “desirable flavors” as a reason for use, stratified by race/ethnicity

**Fig. 4:**
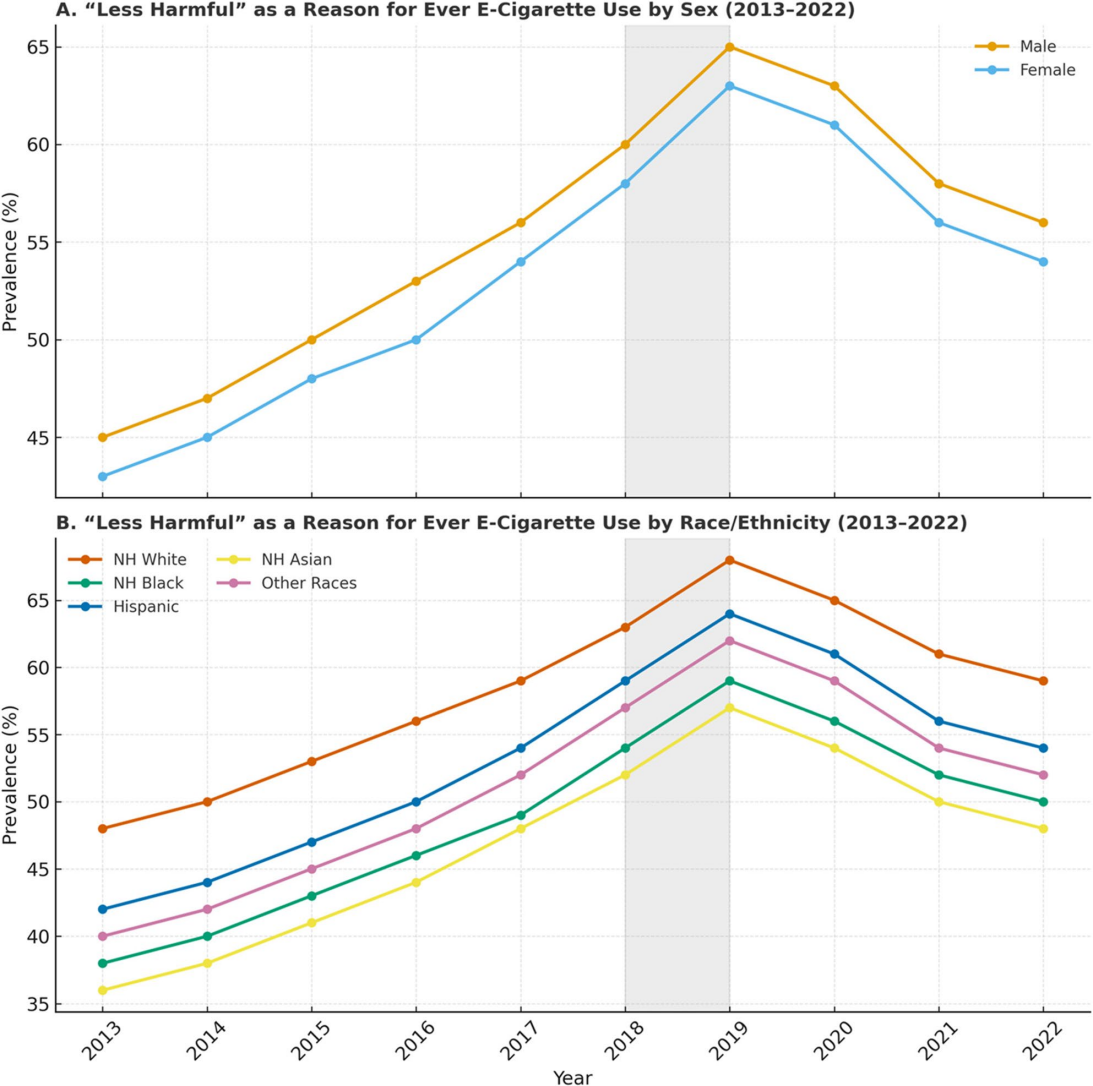
Analysis of the reason “Less harmful than other forms of tobacco” for ever e-cigarette use among U.S. middle and high school students (2013–2022). Panel **A**: Proportion selecting “less harmful than tobacco” as a reason for use, stratified by sex. Panel **B**: Proportion selecting “less harmful than tobacco” as a reason for use, stratified by race/ethnicity

We examined the primary reasons for ever using e-cigarettes, stratified by sex and race/ethnicity (Figs. 2 and 3, and 4). From 2015 to 2022, females consistently showed a higher prevalence of ever using e-cigarettes due to a friend or family member’s usage (Fig. 2). In 2015, 72% of Asian adolescents reported using e-cigarettes because of friend or family use followed by 68% of non-Hispanic Black and 65% of non-Hispanic White adolescents. The highest prevalence of this reason occurred in 2020, with 71.41% of females and 61.64% of males citing it as a reason for use. However, using e-cigarettes due to peers/family use declined among both sexes in 2021 and 2022.

In 2015, the prevalence of e-cigarette use due to desirable flavors was high, with 64% of females and 60% of males reporting it as their reason for use (Fig. 3). Similarly, 65% of Hispanic and 64% of Asian adolescents cited flavors as a reason for use in 2015. However, by 2022, these percentages had sharply declined to 24% among Hispanic and 16% among Asian adolescents. A similar trend was observed across other racial groups, with higher percentages of use in earlier years, followed by a notable decrease in 2021 and 2022. Initially, nearly half of all adolescents used e-cigarettes because they believed them to be less harmful than other forms of tobacco, regardless of sex or race(Fig. 4). However, this belief has declined in more recent years, particularly in 2021 and 2022.

### Ten-year temporal trends and correlates of e-cigarette ever use among U.S. middle and high school students (2013–2022)

Higher odds of e-cigarette ever use were observed among Non-Hispanic Whites, males, those in high school, and cigarette smokers (Table 3). Over the decade, males were 20% more likely to ever use e-cigarettes (AOR:1.20, 95%CI:1.163, 1.242) compared to females. Non-Hispanic Black and Asian adolescents had less than half the odds of using e-cigarettes (AOR:0.49, 95% CI:0.46, 0.52) and (AOR:0.46, 95% CI:0.41, 0.50), respectively, compared to their Non-Hispanic White peers. High school students were more than twice as likely to use e-cigarettes (AOR:2.256,95%CI:2.18–2.33), compared to middle school students. Furthermore, adolescents with a history of cigarette use were significantly more likely to ever use e-cigarettes, with established smokers having 24 times higher odds of ever trying e-cigarettes (AOR: 23.86, 95% CI: 21.84–26.07), compared to those who have never smoked cigarettes (Table 3).

**Table 3:** Logistic regression analysis of the factors associated with e-cigarette ever use among middle and high school students - US (2013–2022)

Covariates Variable	Ever smoking e- cigarette	
	AOR (95% CI)	*P*-Value
Sex		
Female (Ref.)	(Ref.)	(Ref.)
Male	**1.202 (1.163–1.242)**	**< 0.0001**
Race/ethnicity		
NH- White (Ref.)	(Ref.)	(Ref.)
NH- Black	**0.490 (0.464–0.517)**	**< 0.0001**
Hispanic	0.999 (0.962–1.038)	0.9689
Asian	**0.456 (0.413–0.504)**	**< 0.0001**
Other	1.016 (0.957–1.079)	0.5953
School grade		
Middle school (Ref.)	(Ref.)	(Ref.)
High school	**2.256 (2.178–2.336)**	**< 0.0001**
Cigarette use		
Never (Ref.)	(Ref.)	(Ref.)
Experimental*	**9.378 (9.048–9.720)**	**< 0.0001**
Established***	**23.862 (21.843–26.068)**	**< 0.0001**

*Experimental cigarette use = less than 100 days

**Established cigarette use = more than 100 days

*AOR *adjusted odds ratio. The bold values are significant at *p* < 0.05

**Fig. 5:**
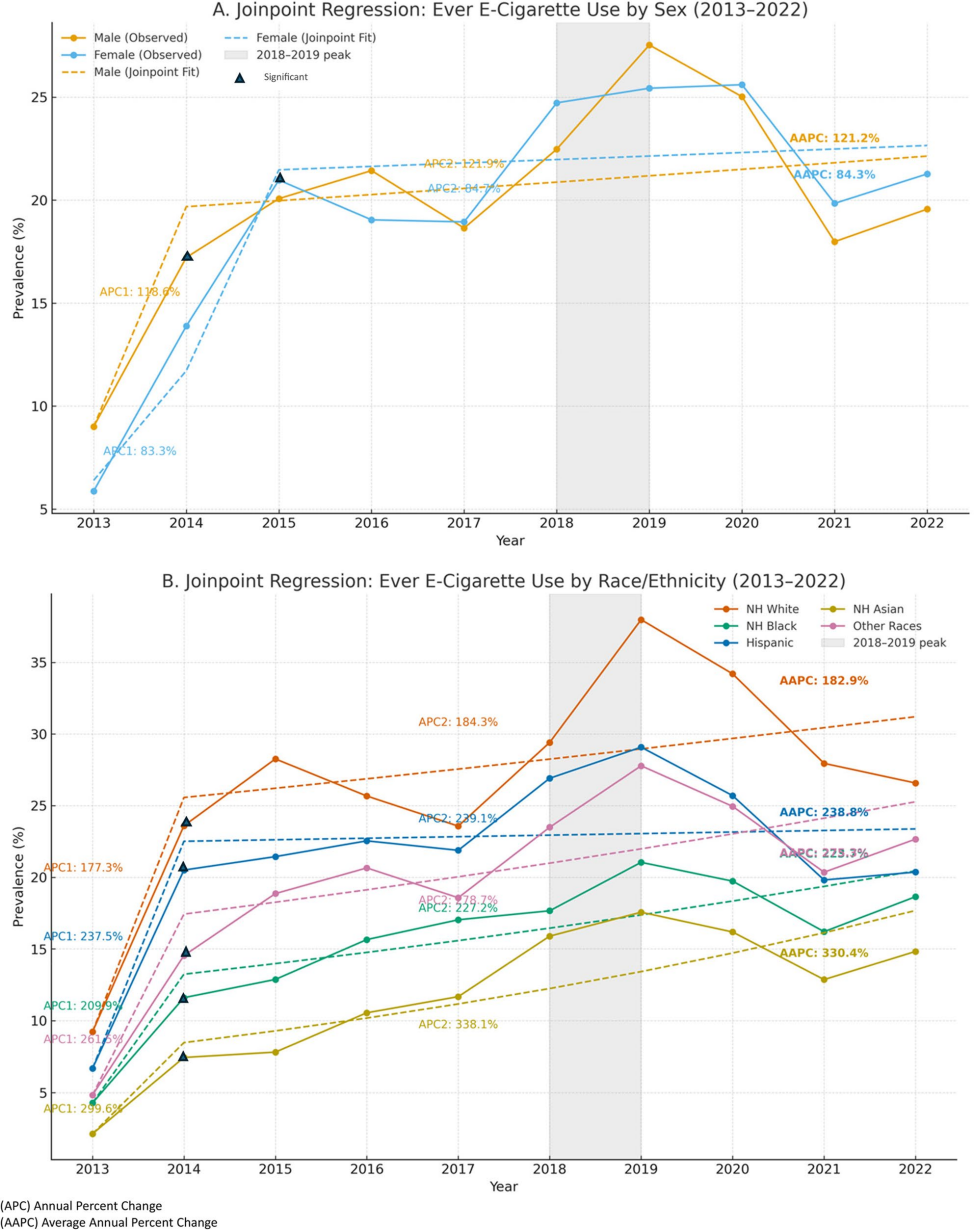
Temporal trends and annual percent change (APC) in e-cigarette ever use among U.S. middle and high school students, stratified by sex and race (2013–2022). Panel **A**: Trends and APC estimates by sex (male vs. female). Panel **B**: Trends and APC estimates by race/ethnicity (Non-Hispanic White, Non-Hispanic Black, Hispanic, Other)

For temporal trends, joinpoint regression model identified a significant increase in ever e-cigarette use among males from 2014 to 2019 and among females from 2015 to 2019 (*p* < 0.05) (Fig. 5). Further, all races demonstrated a significant upward trend after 2014, with the AAPC ranging from approximately 183% in White students to 330% in Asian students, reflecting variability in both the magnitude and timing of increases. While the prevalence of ever e-cigarette use declined between 2019 and 2022 across all demographic subgroups, this decrease was not statistically significant (*p* < 0.05).

## Discussion

The study examined the 10-year trends (2013 to 2022) in e-cigarette use among middle and high school students in the United States. In addition to identifying the primary reasons influencing adolescent e-cigarette ever use, this study evaluates the factors associated with ever having used e-cigarettes within the U.S. adolescent population. E-cigarette use was highest among male, Non-Hispanic White, and Hispanic students. The prevalence of ever e-cigarette use across all demographic subgroups increased steadily until 2019, after which it declined from 2020 to 2022. Moreover, higher odds of e-cigarette ever use were observed among Non-Hispanic Whites, males, high school students, and those who were experimental or established cigarette smokers.

Our study observed that the prevalence among males adolescents who ever used e-cigarettes was slightly higher from 2013 to 2021. However, in 2022, females comprised over half of adolescents who ever smoked e-cigarettes. A similar trend was recently highlighted by the Centers for Disease Control and Prevention (CDC), which reported that more females than males are currently using e-cigarette [1]. A possible explanation for the sex differences in e-cigarette use among adolescents could be targeted product marketing strategies. Initially, e-cigarette marketing was primarily targeted at males, who often purchased these products online, while females tended to obtain them from peers [14]. Additionally, the increase in e-cigarette use among females may be linked to the faster metabolism of nicotine in females due to the presence of estrogen [14]. This faster nicotine metabolism may contribute to poorer smoking cessation outcomes among females [14].

We found that Non-Hispanic White adolescents made up the majority of adolescents who use e-cigarettes from 2013 to 2022. Hispanic adolescents were the next largest group among adolescents who ever used e-cigarettes. Our findings mirrored those reported in a recent review where e-cigarette use was more prevalent among Non-Hispanic White youth and adults compared to Non-Hispanic Black, Hispanic/Latino, and other races [15]. Furthermore, Hispanic youth tend to start experimenting with e-cigarettes at younger ages than those in other racial and ethnic groups [15]. Approximately a third of adolescents who used e-cigarettes in 2013 were established cigarette smokers, however this prevalence dropped to 9% by 2022. This change could be influenced by misconceptions about the health benefits of e-cigarettes, which are often marketed as healthier alternatives and as aids for quitting other tobacco products [19].

Over the past decade, e-cigarette use increased among both males and females, peaking in 2019 at approximately 35% for male and 33% for female adolescents in the U.S. Similar trends were observed across all racial groups. However, amidst the COVID-19 pandemic (from 2020 to 2022), the prevalence of e-cigarette use declined. The pandemic in 2020 appears to have slowed this trend, likely due to changes in the nicotine use behavior caused by stay-at-home orders [20, 21]. Additionally, the NYTS transitioned from paper-and-pencil to electronic administration in 2019, and data collection was conducted online in 2021 due to COVID-19 protocols. Further, adolscents reported difficulties accessing e-cigarettes due to the closure of vape shops and product unavailability [20]. Many adolescents, who typically vaped in social settings or with friends, experienced a reduction in use due to restrictions on social gatherings [20]. Additionally, smoke-free home policies and extended shipping times further contributed to this decline [21]. As a result, a greater number of adolescents who used e-cigarettes self-reported cessation during this period [20].

Our analysis revealed that product use among Non-Hispanic White students doubled from 9.2% in 2013 to 20.5% in 2014, reaching an all-time high of 37.1% in 2019. A similar trend was observed among Non-Hispanic Black students, with use increasing from 4% in 2013 to 14.2% in 2014, and about a quarter of them reporting ever trying the product by 2019. A study published by the *American Academy of Pediatrics* journal supporting the increase in e-cigarette use among youth between 2011 and 2014, with an estimated 2.4 million middle and high school students reporting e-cigarette use in the past 30 days in 2014 [22]. One possible explanation for these trends is that e-cigarettes are not subject to the same marketing and promotion restrictions as traditional cigarettes [23], with, e-cigarette companies able to advertise their products on mass media and other platforms [23]. This unrestricted marketing led to a sharp increase in e-cigarette marketing expenditure, from $3.6 million in 2010 to $125 million in 2014, which contributed to the rapid rise in youth e-cigarette use [23]. By 2014, 68.9% of middle and high school students had been exposed to e-cigarette advertisements from at least one source, with the highest exposure coming from retail stores, followed by the Internet, TV, and movies [24].

Our study further explored the top three reasons for e-cigarette ever use among middle and high school students over the period from 2013 to 2022. The leading reason was the influence of peers or family members who also use e-cigarettes, followed by the availability of flavors, and the ability to use e-cigarettes in areas where traditional tobacco products are prohibited. Our findings are consistent with existing literature. Evidence from a meta-analysis indicated that smoking by family members and friends significantly increases the likelihood of e-cigarette use among adolescents [25, 26]. Flavor availability is a crucial motivator for youth e-cigarette use, with many initiating use with flavored products [27, 28]. Moreover, the minimal residual odor from e-cigarettes, unlike that of traditional tobacco, makes them less detectable, further contributing to their appeal among adolescents [29, 30]. However, numerous policies have been implemented over this decade to address and reduce e-cigarette use among adolescents. The Federal Trade Commission (FTC) recently released its third report on e-cigarette advertising and sales in the U.S. The report underscores initiatives such as self-certification on online orders to ensure users are at least 21 years old and adhere to state laws requiring an adult signature upon delivery of e-cigarette products [31]. Furthermore, in July 2024, the U.S. Food and Drug Administration (FDA) took a significant step by authorizing the marketing of four menthol-flavored e-cigarettes, which are the first non-tobacco flavored e-cigarette products to receive FDA authorization [32]. However, The FDA expressed continued concern over youth e-cigarettes use and stated it will closely monitor the marketing of these products, taking action if companies fail to comply with the marketing guidelines [32].

Our regression analysis revealed that males were 20% more likely to use e-cigarettes compared to females. A similar trend was observed in European regions, where significantly more male students aged 11–17 years used e-cigarettes, particularly among those with medium or higher amounts of pocket money [33]. Non-Hispanic Black and Asian adolescents had less than half the odds of using e-cigarettes, respectively, compared to their Non-Hispanic White peers. Non-Hispanic White youths demonstrated the highest rates of e-cigarette use and a greater preference for flavored products compared to their peers [34, 35]. Conversely, Non-Hispanic Black youths were less likely to use e-cigarettes overall but exhibited higher rates of occasional use and dual use with cigarettes or other tobacco products [35]. Temporal trend analyses further highlight how these disparities have evolved over time. From 2014 to 2019, males experienced a steeper increase in ever e-cigarette use compared to females, reflecting historical marketing patterns that primarily targeted male youth [14]. However, after 2019, prevalence among females stabilized at higher levels, with females comprising more than half of adolescents who ever used e-cigarettes by 2022. This shift suggests a narrowing gender gap, likely influenced by changing marketing strategies, evolving social norms, and increased availability of appealing flavored products.

The current study has several limitations. The primary limitation is that the findings are restricted to descriptive and associations due to the cross-sectional nature of the NYTS data, which limits our ability to examine causal relationships. Additionally, NYTS data are collected from students in schools and may not be generalizable to all adolescents in the US. A key limitation is that the study focused on ever e-cigarette use and did not address use frequency, which may be explored in future research. The survey did not assess the adolescent’s pocket money availability, as other studies have revealed that an increase in income may be associated with higher e-cigarette use [33, 36]. Additionally, the survey did not evaluate larger macro-level factors such as the impact of COVID-19 or the several policy changes. However, the strengths of this study lie in its examination of trends in e-cigarette use among adolescents in the United States from 2013 to 2022, making it one of the few studies to analyze large national data across that decade [37]. As well as its nuanced investigation of the underlying reasons for use. Additionally, the survey is a collaborative effort between the CDC and the FDA, ensuring robust oversight and methodology. Future longitudinal studies should provide a clearer understanding of e-cigarette use habits and the motivations for use among adolescents in the US.

## Conclusion

This study provides an overview of e-cigarette use trends among U.S. middle and high school students from 2013 to 2022. E-cigarette use increased steadily, peaking in 2019 before declining amides the covid-19 pandemic. Non-Hispanic White adolescents represented the largest proportion of adolescents who used e-cigarettes, with a growing proportion of Hispanic adolescents over time. The decline in e-cigarette use among adolescents who smoke cigarettes and the rise among those who have never smoked traditional cigarettes suggest a shifting pattern of use. Key motivators for e-cigarette use included social influences and flavored products, though these reasons became less significant over time as perceptions of harm changed. These findings highlight the urgent need for targeted public health interventions and sustained policy regulations to effectively address youth e-cigarette use.

### Financial disclosures

Non were reported by the authors of this paper.

## Data Availability

No datasets were generated or analysed during the current study.
